# Developing an Intervention and Evaluation Model of Outdoor Therapy for Employee Burnout: Unraveling the Interplay Between Context, Processes, and Outcomes

**DOI:** 10.3389/fpsyg.2022.785697

**Published:** 2022-03-03

**Authors:** Roald Pijpker, Esther J. Veen, Lenneke Vaandrager, Maria Koelen, Georg F. Bauer

**Affiliations:** ^1^Health and Society, Social Sciences Group, Wageningen University, Wageningen, Netherlands; ^2^Urban Food Issues, Aeres University of Applied Sciences, Almere, Netherlands; ^3^Center of Salutogenesis, Division of Public and Organizational Health, Epidemiology, Biostatistics and Prevention Institute, University of Zurich, Zurich, Switzerland

**Keywords:** burnout, context, evaluation, intervention, nature, resources, salutogenesis

## Abstract

**Background:**

Burnout is a major societal issue adversely affecting employees’ health and performance, which over time results in high sick leave costs for organizations. Traditional rehabilitation therapies show suboptimal effects on reducing burnout and the return-to-work process. Based on the health-promoting effects of nature, taking clients outdoors into nature is increasingly being used as a complementary approach to traditional therapies, and evidence of their effectiveness is growing. Theories explaining how the combination of general psychological support and outdoor-specific elements can trigger the rehabilitation process in outdoor therapy are often lacking, however, impeding its systematic research.

**Aim:**

The study aims to develop an intervention and evaluation model for outdoor therapy to understand and empirically evaluate whether and how such an outdoor intervention may work for rehabilitation after burnout.

**Methodological Approach:**

We build on the exemplary case of an outdoor intervention for rehabilitation after burnout, developed by outdoor clinical psychologists in Netherlands. We combined the generic context, process, and outcome evaluation model and the burnout recovery model as an overarching deductive frame. We then inductively specified the intervention and evaluation model of outdoor therapy, building on the following qualitative data: semi-structured interviews with outdoor clinical psychologists and former clients; a content analysis of the intervention protocol; and reflective meetings with the intervention developers and health promotion experts.

**Results:**

We identified six key outdoor intervention elements: (1) physical activity; (2) reconnecting body and mind; (3) nature metaphors; (4) creating relationships; (5) observing natural interactions; and (6) experiential learning. The results further showed that the implementation of these elements may facilitate the rehabilitation process after burnout in which proximal, intermediate, and distal outcomes emerge. Finally, the results suggested that this implementation process depends on the context of the therapist (e.g., number of clients per day), therapy (e.g., privacy issues), and of the clients (e.g., affinity to nature).

**Conclusion:**

The intervention and evaluation model for outdoor therapy shows how key outdoor intervention elements may contribute to the rehabilitation process after burnout. However, our model needs to be further tested among a larger group of clients to empirically evaluate whether and how outdoor therapy can support rehabilitation.

## Introduction

### Employee Burnout and Rehabilitation Programs

Burnout is defined as “a work-related state of exhaustion that occurs among employees, which is characterized by extreme tiredness, reduced ability to regulate cognitive and emotional processes, and mental distancing. These four core dimensions are accompanied by depressed mood as well as by non-specific psychological and psychosomatic distress symptoms” (Schaufeli et al., 2020, p. 28). Being burned-out reflects a process of not being able to work due to chronic exhaustion and impaired cognitive functioning (i.e., I cannot do my job anymore), and a process in which employees mentally or even physically distance themselves from their jobs to prevent further depletion (i.e., I do not want to do my job anymore; Schaufeli et al., 2020). Over time, burnout adversely affects both employee health and wellbeing, such as an increased risk for cardiovascular diseases (Appels and Schouten, 1991), as well as affecting organizational performance, including decreased levels of job satisfaction and increased absenteeism (Alarcon and Edwards, 2011; Ruitenburg et al., 2012; Salvagioni et al., 2017). In the Netherlands, burnout is the most significant predictor of high sick leave and replacement costs, estimated to be 3.1 billion euros annually (TNO, 2020).

The so-called Dutch “rehabilitation guidelines” emphasize facilitating the return-to-work (RTW) process through reducing burnout complaints, using cognitive behavioral therapies (CBT) and psychoeducation, combined, if possible, with intervening in the working context (van Dam et al., 2017; Van Dam, 2021). However, evaluations of the effectiveness of such burnout rehabilitation interventions reveal suboptimal results in terms of both reducing burnout complaints and facilitating the RTW process (Awa et al., 2010; Ahola et al., 2017; Perski et al., 2017; Pijpker et al., 2020); for example, Blonk et al. (2006) showed that combining both CBT and a reduced workload only facilitated a partial RTW but did not fully alleviate burnout complaints. This is in line with the findings from de Vente et al. (2015), who showed that recovering from burnout complaints and the RTW are two relatively independent processes, which both remain poorly understood. As Demerouti et al. (2021) recently synthesized, theoretically grounded and evidence-based best-practice rehabilitation interventions are simply lacking in burnout literature. The evidence base surrounding existing burnout rehabilitation interventions is thus weak (Pijpker et al., 2020; Schaufeli, 2020), as acknowledged in the rehabilitation guidelines currently applied in the Netherlands (Van Dam et al., 2017).

### Outdoor Therapy Interventions for Employee Burnout

While the effects of existing burnout rehabilitation programs are suboptimal, evidence supporting the positive effects of nature on our health and wellbeing is accumulating (Hartig et al., 2014). Nature exposure has a range of positive physiological effects, such as reducing the levels of salivary cortisol and blood pressure (Twohig-Bennett and Jones, 2018); beneficial psychological effects, including increased self-esteem and an improved ability to concentrate (Bratman et al., 2012); and overall improved health and wellbeing (White et al., 2019). Besides the effects of nature exposure, undertaking activities in nature, such as walking or gardening, reduces stress-related complaints and enhances overall health and wellbeing (Annerstedt and Währborg, 2011; Sahlin et al., 2014).

Besides the positive effects of nature exposure on our general health and wellbeing, nature exposure also supports recovery from acute and chronic stress. For example, a recent meta-analysis showed that exposure to natural environments leads to significant more stress reduction, using both objective (e.g., salivary cortisol) and subjective (e.g., self-reported stress) measures, compared to built environments (Yao et al., 2021). Concerning nature exposure in relation to recovery from burnout complaints, Cordoza et al. (2018) showed that taking daily work breaks among nurses in a garden compared to indoor-only breaks resulted in significantly less burnout complaints.

Based on the health-promoting and restorative effects of nature, taking clients outdoors into nature is increasingly being used as a complementary approach to traditional therapies for various client and patient groups (see Annerstedt and Währborg, 2011; Sahlin et al., 2014, 2015), generally called “outdoor therapy” (Cooley et al., 2020). In the present study, we define outdoor therapy as the combination of providing psychological support used in traditional therapies and specific outdoor elements with the aim of supporting the rehabilitation process after burnout, facilitated by a licensed practitioner (Annerstedt and Währborg, 2011; Cooley et al., 2020). For employee burnout, psychological support used in traditional therapy for burnout entails CBT and psychoeducation (Van Dam et al., 2017).

### Scientific Lacuna and Study Aim

Although evidence for the effectiveness of outdoor therapies in tackling employee burnout is emerging (Stigsdotter and Grahn, 2003; Sahlin et al., 2014; Stigsdotter et al., 2018; van den Berg and Beute, 2021), theories or models explaining how they can facilitate the rehabilitation process after burnout are often lacking (Annerstedt and Währborg, 2011). Recently, in their systematic literature review focusing both on outdoor therapist and client experiences, Cooley et al. (2020) developed a best-practice framework for key considerations for taking therapy outdoors in general. This framework shows various therapy approaches and natural environments used in the outdoors, as well as potential practical, therapeutical, and organizational issues and solutions when bringing conventional therapy outdoors (Cooley et al., 2020). Outdoor therapies are considered “complex interventions,” however, meaning that the implementation of various intervention elements depends on the specific context in which the intervention takes place (Annerstedt and Währborg, 2011, p. 384).

In order to understand how outdoor therapies work for employee burnout, it is first important to describe how intervention elements in combination with the underlying intervention context may facilitate rehabilitation after burnout. To our knowledge, no attempts have been made to unravel how key elements of outdoor therapy in combination with the underlying intervention context may facilitate the rehabilitation process after burnout. Therefore, this study aims to develop an intervention and evaluation model for outdoor therapy that helps understanding whether and how such outdoor interventions may work for rehabilitation after burnout. It is important to note that we are not conducting an effect and process evaluation of outdoor therapy for employee burnout. Rather, this explorative study will empirically develop how such an outdoor intervention may work for employee burnout, without testing or confirming the assumptions among a large representative group of clients.

## Materials and Methods

We build on the exemplary case of an outdoor intervention for rehabilitation after burnout developed by two outdoor clinical psychologists in Netherlands (in Dutch: “De Buitenpsychologen”).^1^ They developed and implemented a training program for clinical psychologists who want to bring their therapy outdoors to treat employee burnout and other psychological disorders, and thereby become a licensed outdoor therapist. As outdoor therapy is not an accepted therapy intervention in mainstream mental healthcare settings, almost all outdoor therapists are self-employed; however, these practitioners are allowed to diagnose employees with burnout or other psychological disorders. This means that Dutch healthcare insurers can financially cover outdoor therapy for their clients.

To develop the intervention and evaluation model of outdoor therapy, we build on the generic context, process, and outcome (CPO) evaluation model (Fridrich et al., 2015) and the generic burnout recovery model (BRM; Pijpker et al., 2021; Van Dam, 2021) as an overarching frame. Based on this deductively derived generic frame, we then inductively specified the intervention and evaluation model of our exemplary outdoor therapy case in Netherlands, using both primary and secondary data. Before describing the data collection and analysis procedures, we will first introduce the CPO and BRM models.

### The Generic CPO Evaluation Model Applied to Outdoor Therapy for Employee Burnout

The generic CPO evaluation model is increasingly being used to explore how the implementation of an intervention in a certain context can facilitate change processes among participants, after which certain proximal, intermediate, and distal outcomes emerge (Fridrich et al., 2015). Using our exemplary case in the Netherlands, we aimed to unravel how the implementation of therapy outdoors may facilitate the rehabilitation process after burnout. We also aimed to identify indicators to empirically evaluate whether and how the intervention works. *Context* can be divided into an omnibus context, which refers to the general and implementation setting, and a discrete context, which refers to situationally specific variables (Fridrich et al., 2015). With regard to outdoor therapy in the Netherlands, the omnibus context refers to the location of the therapy or the season in which it takes place. The discrete context refers to aspects directly relevant to the implementation process of key outdoor intervention elements; for example, clients can be in different phases of the rehabilitation process (Pijpker et al., 2021; Van Dam, 2021), which may require that outdoor elements are adapted to achieve therapeutic goals in a certain rehabilitation phase.

Concerning the *change process*, two subcategories can be distinguished (Fridrich et al., 2015): (1) the implementation process of key (outdoor) intervention elements, and (2) how the implementation of intervention elements facilitates the rehabilitation process after burnout. This change process inevitably results in proximal, intermediate, and distal *outcomes* (Fridrich et al., 2015), such as increasing clients’ feeling of control over their rehabilitation (Pijpker et al., 2021; Van Dam, 2021). Finally, the model distinguishes three different phases: the preparation phase (preparing the intervention), the action cycle phase (conducting the intervention), and the appropriation phase (after the intervention; Fridrich et al., 2015). In our study, the emphasis was placed on the preparation and action cycle phases to unravel the interplay between the context, processes, and outcomes of outdoor therapy for employee burnout.

### The BRM

To understand how outdoor therapy may facilitate the rehabilitation process after burnout among employees, we will use the generic BRM (Pijpker et al., 2021; Van Dam, 2021). This model is based on the theory of salutogenesis, originally proposed by Antonovsky (1979, 1987). Salutogenesis aims to explain the factors affecting health and wellbeing in the everyday life context, and complements the pathogenic approach which focuses on factors causing disease and infirmity (Mittelmark and Bauer, 2017). The central concept of salutogenesis is the sense of coherence (SOC), which reflects the extent to which people experience life as comprehensible, manageable, and meaningful (Eriksson, 2017).

People with a strong SOC feel confident to use internal and external resources, enabling them to successfully cope with stressors, such as daily hassles or significant life events. In the present study, burnout is defined as a life event stressor, as it is characterized by feelings of losing control over one’s working situation and resources (Bernier, 1998; Fjellman-Wiklund et al., 2010; Salminen et al., 2015; Pijpker et al., 2021). Resources that enable successful coping with stressors are called generalized resistance resources (GRRs), which can be internal, such as high levels of self-esteem, and external, such as having good social relationships with one’s colleagues (Idan et al., 2017).

Recently, Pijpker et al. (2021) applied the theory of salutogenesis to explore the processes underlying a successful recovery after burnout. The BRM was developed based on multiple in-depth interviews with burned-out employees, focusing on their overall recovery experiences within and beyond the therapeutic context of outdoor therapy. The model consists of four generic recovery phases: (1) facing the crisis; (2) addressing root resources and stressors; (3) seizing and realizing the opportunity; and (4) staying in work. These phases represent a variety of GRRs (see Table 1). Additionally, social support (e.g., from family, colleagues, therapist) and regaining a feeling of control are two overarching GRRs facilitating the rehabilitation process. With regards to the CPO evaluation model, the four rehabilitation phases reflect the change process (Pijpker et al., 2021; Van Dam, 2021), facilitated by the implementation of key outdoor intervention elements in the underlying context.

**Table 1:** Burnout recovery model including key treatment goals and generalized resistance resources (based on: Pijpker et al., 2021; Van Dam, 2021).

Recovery phase	Generalized resistance resources
Facing the crisis *Treatment goal:* *enabling clients to accept the situation and that change is required*	Accepting the problem Resting Reducing stressors in work/private life Financial support from social security system* Psychological support
Addressing root stressors and applying resources *Treatment goal: reducing clients (physiological) stress levels and restoring the connection between body and mind*	Relaxing exercises Mindfulness exercises Daily structure Physical activity Experiencing nature Feeling physically and mentally well
Seizing and realizing the opportunity *Treatment goal: empowering client to apply new skills and coping strategies to make change happen*	New coping strategies Reflecting on key stressors and resources Social support Connectedness with the working context Approving one’s feelings Courage
Staying at work *Treatment goal: strengthening client’s capacities to maintain a sustainable and meaningful working life.*	Confidence in the future Awareness of potential pitfalls Meaningfulness in work/private life

* In the Netherlands, social security systems like healthcare insurance cover treatment for people on sick-leave who suffer from burnout. Also, employers are responsible for paying wages during the first two years of sick-leave (Schaufeli, 2017).

### The Collection of Related Primary and Secondary Data

Both primary data sources (i.e., interviews with former clients, outdoor therapists, intervention developers, and experts) and secondary data sources (i.e., the existing intervention protocol) were used to specify the intervention and evaluation model of outdoor therapy. Participants were selected by means of purposive sampling (De Vaus, 2001), which was used to ensure that the outdoor therapists all had the same training and expertise related to this therapy and that clients had fully participated the outdoor intervention and successfully recovered from burnout. We employed online semi-structured interviews with outdoor therapists (*N* = 4; 4 females; age range, 32–51) to understand their practice and experiences with outdoor therapy. The questions focused on how the combination between general psychological support and specific outdoor elements may work for employee burnout. More specifically, we asked for the key outdoor intervention elements they applied, and for practical examples of implementing these key elements. During the interview, the BRM was used to discuss how the key intervention elements can trigger change processes and outcomes in each burnout recovery phase. Interviews with former clients (*N* = 4; 2 males, 2 females; age range, 24–46) were conducted to enrich our insight into how outdoor therapy fits with the four recovery phases of the BRM (Pijpker et al., 2021; Van Dam, 2021). Burnout was diagnosed as being on sick leave or not able to perform work-related tasks for at least 6 months due to work-related stressors and ruling out any other psychological disorders (Van Dam et al., 2017). The interviews started with an open question about the experiences of participating in outdoor therapy, after which the BRM was used to understand the experiences about their rehabilitation as a result of receiving outdoor therapy.

Besides interviews with therapists and clients, we used the intervention protocol of outdoor therapy for employee burnout as secondary data to identify key intervention elements and their implementation processes. Finally, we held online reflective meetings with experts in the field of health promotion (*N* = 6, two sessions total) and the Dutch developers of outdoor therapy (*N* = 2, five meetings total). The overarching aim of these reflective meetings was to explore and clarify the overall development of the intervention and evaluation model of outdoor therapy, thereby creating multiple feedback loops for specifying the final model.

### Data Analysis

All interviews were transcribed verbatim. The key points of the reflective dialogues were summarized from the recordings. The analysis entailed an iterative process of inductively identifying key outdoor intervention elements, how the implementation of these elements may facilitate the rehabilitation process, and which outcomes may emerge. Besides describing the context, change processes, and outcomes of outdoor therapy, we also focused on identifying the contextual factors related to the implementation of the key outdoor intervention elements.

The transcripts and the intervention protocol were read several times to enhance familiarity with the data (Corbin and Strauss, 1990). Using ATLAS.ti (version 9.0), the data were then open-coded line-by-line with no rules about what should be labelled and how; for example, labels could be descriptive or conceptual. Following the open-coding of all materials, the codes were connected, linked, and grouped into themes (axial coding), after which the first author allocated themes to key outdoor intervention elements, the four rehabilitation phases, outcomes, and implementation-related indicators of the key outdoor elements (selective coding). This analysis procedure was repeatedly discussed with all authors and the findings were reflected upon with the intervention developers to enrich the results. By doing so, we used the generic principles of thematic analysis (Braun and Clarke, 2006), which was deemed appropriate to inductively identify the aspects within the generic deductively derived CPO and BRM frame. Notes and audio recordings from the reflective meetings were used to further support the selective coding process (for example, distinguishing proximal and intermediate outcomes). This process was repeated until data saturation was achieved.

### Ethical Considerations

The methodology for this study was approved by the Social Sciences Ethics Committee (Wageningen University and Research) on June 13, 2019. Prior to the interviews, the first author emailed the participants to inform them about the aims of the study. The researchers were aware that interviews with former clients involved a potential risk of traumatization by asking people to revisit what they had experienced before. The participants were informed about this possibility and were told that they could contact the interviewer and occupational doctor at any time after the interview. All participants provided signed informed consent before the interviews took place. Since all interviews took place online due to the COVID-19 restrictions, they again provided consent before starting the interview.

## Results

### The Intervention and Evaluation Model for the Outdoor Therapy of Employee Burnout

**Figure 1:**
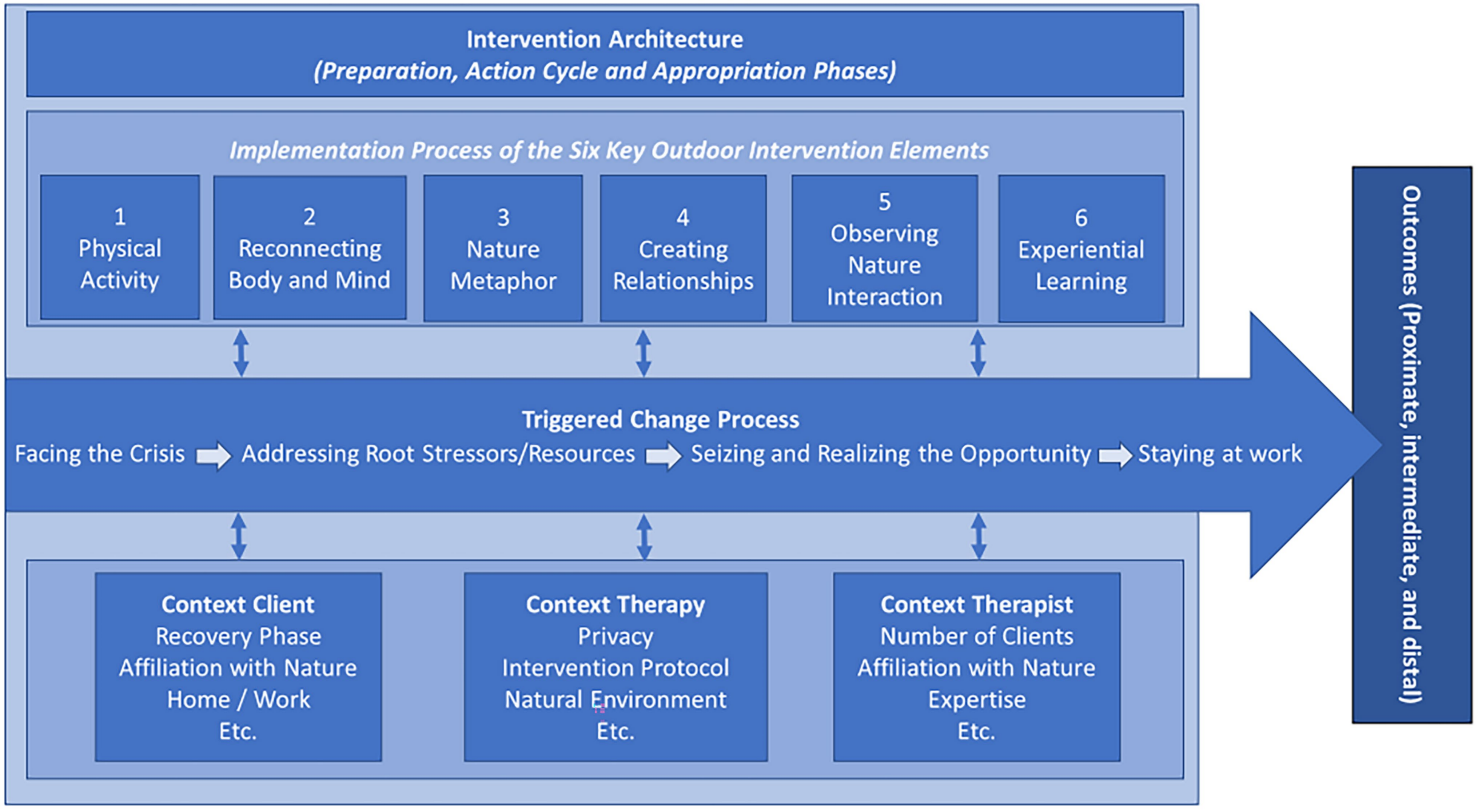
Intervention and evaluation model of outdoor therapy for employee burnout.

Figure 1 shows the intervention and evaluation model for outdoor therapy as an overarching outcome of our data analysis. The model presents how the implementation of key outdoor intervention elements may facilitate the rehabilitation process after burnout. This implementation process strongly depends on the discrete context of the: 1) therapist, 2) client, and 3) therapy. We first describe the results related to the implementation of key outdoor intervention elements, as well how these elements may facilitate the rehabilitation process and outcomes. Finally, we present our findings concerning the role of the discrete context in implementing the key outdoor intervention elements, as well as the context-related indicators of these elements.

### Key Outdoor Intervention Elements

The analysis showed that outdoor therapy involves a maximum of 12 sessions per client per year. The preparation phase (sessions 1–3) covers the intake, including exploring therapeutic goals and discussing mutual expectations from both the client and the therapist (IP6:64).^2^ The action cycle phase (sessions 4–8) facilitates the rehabilitation process (IP6:65). The sessions in the appropriation phase (sessions 9–12) serve as a backup in case a client drops out of work again, which might indicate more complex psychological problems than burnout (IP6:66).

The analysis showed that the standard CBT and psychoeducational approaches are combined with six key outdoor intervention elements. These intervention elements are: (1) physical activity, (2) reconnecting body and mind, (3) nature metaphors, (4) creating relationships, (5) observing natural interactions, and (6) experiential learning. The analysis further showed that the selection and implementation of intervention elements are not predefined to belong to a certain therapy session or GRR in the rehabilitation phases. Rather, they can be applied in any session to facilitate change in single or multiple GRRs in single or multiple rehabilitation phases. We now describe the implementation of the six key outdoor intervention elements and how, in our analysis, they were found to facilitate the rehabilitation process after burnout.

### The Implementation and Rehabilitation Process

The first key outdoor intervention element is *physical activity*, meaning that therapists walk with clients in the outdoors. By doing so, therapists aim to improve the client’s physical health and mental wellbeing, which are GRRs in the second rehabilitation phase. They also encourage clients to walk outside every day for half an hour or 1 h, thereby using physical activity to create a daily structure and prompting the client to make a change in their rehabilitation. Daily structure is a GRR in the second rehabilitation phase, and activating the client relates to the overarching GRR in the rehabilitation process: regaining a feeling of control. To achieve this, therapists give homework exercises to clients containing daily outdoor walks and also reflect on their experiences of walking daily, sometimes by means of psychoeducation to clarify what clients are feeling. Therapists observe that by going outdoors and being physically active, clients experience the success of having achieved something that day and feel at ease, which also relates to the GRR of regaining a feeling of control over the rehabilitation process.

“*[Clients report] ‘I actually went outside today and I did go for a walk, which I can check off my to-do list.’ … ‘Nice that I have done this today’ … That makes them relaxed*.” (ID5:34)

Therapists also observe that clients share their story more easily while walking, as being physically active stimulates the creative part of their brain, which results in “deeper” conversations (ID5:16). Therapists then use CBT and psychoeducation to clarify the client’s emotions, feelings, beliefs, and thoughts, thereby addressing both the second and third rehabilitation phases (e.g., GRR: approval of one’s feelings). This observation was also reflected by clients.

“*I think because of walking, my brain, thoughts, body are ‘turned on’, so it is easier to change thoughts and beliefs and share things.*” (CL2:18)

Besides the direct effects of being physically active, all therapists shared that observing how clients walk is an opportunity to observe their body language. Most clients tend to walk or “race” during the therapy sessions. This is often directly used in the therapy sessions through psychoeducation and CBT; for example, by reflecting on the clients’ walking pace. By literally slowing the client down, the therapists observe clients feel more relaxed and aware of their feelings, which is a GRR in the second rehabilitation phase.

*“Walking too fast is actually a beautiful metaphor. I ask clients if they also walk two meters in front of their colleagues at work or other people. Then I try to talk to them, and I notice that they are not able to talk with me normally and are out of breath. Again, I ask them if they are in front of everyone even when out of breath* … *What would happen if you walked one gear slower?”* (OT2:15)

Although all therapists and most clients reported the beneficial effects of physical activity on the rehabilitation process, one client reported that walking, especially in the first couple of sessions, was more a burden than helping him (CL1:17).

The second key outdoor intervention element is *reconnecting body and mind*, meaning that the direct effects of just being in nature are combined with sensory or mindfulness exercises. Therapists reported often seeing burnout clients lost in their own thoughts. Before using CBT or psychoeducation, therapists emphasize that it is important to slow down, restore the connection with how they feel in the present moment, and let go of previous thoughts (IP:20). Just being in nature was reported to slow clients down and restore their connection with how they feel in the present moment, without any additional therapy work. This relates to the first rehabilitation phase (e.g., GRR: resting). When they apply sensory or mindfulness exercises in nature, they notice that clients are able to let go of previous thoughts, feelings, and emotions, which relates to the third rehabilitation phase (e.g., GRR: approval). Being outdoors therefore enables clients to become a “mindful owner” of their thoughts and lets them experience what being in the present moment can mean for their health and wellbeing (IP:6). By doing so, clients enhance their GRR, regaining a feeling of control over their rehabilitation.

“*I slow down the walking pace on purpose and tell clients to be silent and to focus on what they see, smell, hear, feel, and taste for 10 min. Their attention shifts from their minds to the present moment.*” (OT3:20).

Therapists shared that they can directly observe when a client is in the present moment; clients breathe more deeply, sweat less, and have less tense shoulders when being present in nature. If deemed appropriate, the therapists use psychoeducation to clarify to the client what their feelings mean, enabling the clients to approve their feelings and emotions (i.e., GRR in the third rehabilitation phase). Although most clients shared the therapists’ observations, one client commented that it takes time to learn and experience what reconnecting the body and mind can bring to their rehabilitation, which relates to the second and third rehabilitation phases.

“*I had the feeling that the therapist thought my mind and body should calm down first. …. But why would I do these breathing exercises? That was really weird in the beginning. Alright, I thought, let us do it anyway. But that was not what I expected from a therapist. … Far later, I realized and experienced that my incorrect breathing turned out to be an important part of the puzzle to alleviate stress.*” (CL1:33)

The third key outdoor intervention element is **nature metaphors**, meaning that natural environments are used as a mirror for reflection to achieve certain therapeutic goals in the rehabilitation process. Reflection is a GRR in the third rehabilitation phase. Nature metaphors were reported by therapists to be very useful in order to sustain and visualize verbal communication, enabling clients to better understand or express feelings, thoughts, behavior, desired goals or changes, or the concepts used in CBT or psychoeducation. Clients emphasized that nature metaphors helped them to express how they feel in the present moment compared to the past, but also what they would like to achieve in the rehabilitation process. This relates to the overarching GRR of regaining a feeling of control over the rehabilitation process.

*“You ask the client to point to something in nature that matches their story or feeling. You can also use the weather or natural elements to clarify abstract theoretical concepts in CBT or psychoeducation; for example, burned-out clients find it very logical that a tree requires food, light, protection, and other vital resources, but forget they need those as well.*” (OT3:24)

“*I remember I had to express how I feel by standing between two trees on a scale from one to ten, and where I would like to be next time.* … *We did the same by taking pictures of bushes to express how I felt and which bush I would like to be*.” (CL4:10)

The fourth key outdoor intervention is **creating relationships**, meaning that the outdoors facilitates the development of the relationship between the client and their therapist in several ways. Most clients and therapists reported their relationship to be strong, because walking or sitting next to each other supports a natural flow in the conversation, allowing silences without putting the pressure on the client to answer immediately (ID5:22). Additionally, nature is experienced as something “shared,” making both the client and therapist aware of being part of something bigger; the clients think of themselves less as “the client” and the therapist feels less of “the professional.” Most clients contend that their therapist is “walking with them in the conversation,” meaning they do not merely talk based on a model, but really listen and give tailored advice about what clients can do or how they can look at things differently. This relates to all four rehabilitation phases, but in particular the two overarching GRRs: regaining feelings of control over the rehabilitation process and experiencing social support.

“*Talking next to each other outdoors is less confronting. I felt like I had so much ‘space’ to tell my story. … She literally gave me the space, which made me feel so relaxed. … These were very inspiring talks, which I did not expect. I thought she would tell me what to do, like create a structure etc. … but not at all. We talked about essential things for my recovery process, about meaningfulness in life beyond work; it was a very deep conversation. … No ‘do this, do that’ kind of bullshit, which anyone can find in books about burnout or at any general practitioner.*” (CL3:9)

One outdoor psychologist and one client reported diverging perspectives. The therapist shared that one of her previous clients was not always able to make eye contact while walking (OT1:21). The client emphasized that the relationship with the therapist was indeed mutual, but did not attribute any influence of nature on their relationship (CL1:64).

“*I had one client who experienced not looking at each other as terrible, as she wanted a lot of confirmation regarding my thoughts about what she said* … *I solved it by sitting on a bench with her, 1.5 meters apart due to the COVID-19 measures*.”

“… *I was like a turtle lying on my back and needed professional help. I was very skeptical about the idea of outdoor therapy. I really had a perfect connection with my therapist, but being outdoors did not contribute to that relationship at all.*”

The fifth key outdoor intervention element is *observing nature interaction*, meaning that the way in which the client interacts with the natural environment informs the therapist in various ways; for example, therapists can observe body language to assess whether someone is in the present moment by observing if clients notice what is happening around them. By doing so, therapists can reflect with the clients on their feelings, behaviors, and thinking using CBT or psychoeducational approaches (IP6:48), which relate to various GRRs in all rehabilitation phases. However, therapists cannot always intentionally plan on observing nature interaction, as clients are sometimes suddenly triggered by what they see or what is happening in nature.

“*Then we arrived at a path with a fly agaric with no other things around it.* … *She became very emotional suddenly and started crying very hard, while I was thinking how on Earth can I access her feelings. The client explained that she was very lonely and that rather than seeing a beautiful fly agaric, the mushroom was representing her feelings*.” (OT1:27)

The sixth key outdoor intervention element is **experiential learning**, meaning that natural elements are used to achieve certain therapeutic goals in the rehabilitation process; for example, trees or bushes are used to help clients to experience and learn to set limits, position themselves in relation to colleagues or social networks, or worry less. By doing so, clients experience what it could take to achieve their desired changes and how coping strategies, such as setting limits at work, can help them to remain in work. By doing so, the GRRs in the third and fourth rehabilitation phases are addressed (e.g., the GRR of courage to make changes at work).

“*The focus on returning to work can be internalized by the ‘past-present-future’ exercise in nature. The client has to select three trees that reflect where he or she is coming from, currently is, and where the desired outcome is. By physically standing still at the three situations, the client experiences and learns about the past, present, and future.*” (IP6:42)

### The Role of the Discrete Context

Our analysis showed that the implementation of key outdoor intervention elements, and hence the change processes, strongly depends on the discrete context. We identified three different discrete contexts, namely, the context of the client, of the therapist, and of the therapy.

Concerning the *context of the client*, most clients had an affiliation with or preference for receiving therapy outdoors, mainly because they like walking or have negative experiences with traditional indoor therapies. Clients also differed in their rehabilitation phases. For some clients, learning to deal with the working environment was key in the rehabilitation process, whereas for other clients, changes in their private life were experienced as key factors.

“*Based on previous experiences with indoor psychologists and the general practitioner, my expectations were not that high to be honest. I thought all these conversations would probably not help me, so I just brought my dog to the outdoor therapy sessions. If I indeed had not found it helpful, I at least went for a nice walk with the dog, which was a major reason for joining outdoor therapy… I was really surprised it all worked out so well as they took my previous experiences seriously*…*They really listened to my story and did not just tell me what to do from a theoretical model.*” (CL3:6)

With regards to the *context of the therapist*, several therapists said that they are often self-employed, meaning that they have a lot of autonomy compared with those using highly standardized protocols and administration processes in indoor mental healthcare settings. They also reported different preferences for specific types of natural environment and natural exercises, which directly influences how intervention elements are implemented. Finally, all shared a strong affiliation for being outdoors, and made the “step outside” for their own health and wellbeing first, as they were experiencing high amounts of stress working indoors.

“*I see working outdoors as playing outdoors to be honest. There is a completely different ‘loading’, which makes it all less heavy than working indoors.*” (OT4:15)

Finally, the *context of the therapy* itself relates to aspects about opportunities in the outdoor environment and the types of natural places used; for example, living in a metropolitan area offers fewer chances to go to a large remote forest. Other characteristics relate to how to work within COVID-19 measures and rules around ensuring privacy. Finally, the intervention protocol was argued to be used as a decision tool, and not as a one-size-fits-all approach.

“*I think you can talk about a decision tree, rather than an intervention protocol…You first have to really listen to the client and then choose different exercises, which will facilitate a certain therapeutic goal…It is always about tailoring the needs of the client to the possibilities within the natural environment and the capacities of the therapist.*” (ID5:37)

Table 2 shows the context indicators of the client, therapy, and therapist, which were identified based on the analysis of the intervention protocol, interviews with clients and therapists. All these factors in the discrete context influence how the key outdoor intervention elements are implemented.

**Table 2:** Context indicators of the client, therapy, and therapist.

Client	Therapy	Therapist
Home resources/demands	Influence of nature on achieving therapeutic goals	Affinity with nature
Work resources/demands	Satisfaction with the therapy	Number of clients per day
Rehabilitation phase	Satisfaction with the therapist	Experience with outdoor therapy
Affinity with nature	Type of natural environment used	
Previous experiences with therapy	Use of intervention protocol	
Participation in other rehabilitation programs	Number of therapy sessions	
Changes observed by significant others	Duration of therapy sessions	
Social support from employer		
Social support from occupational doctor		

### Outcomes

Table 3 presents the proximal, intermediate, and distal outcomes as a result of the implementation of the six intervention elements and triggered change process, based on our analysis of the experiences from therapists, clients, and intervention initiators. *Proximal outcomes* are becoming a mindful owner of one’s thoughts, becoming physically active, feeling relaxed, and experiencing success. *Intermediate outcomes* are regaining a feeling of control over the rehabilitation process, applying new pro-active coping strategies, feeling meaningfulness in (working) life, feeling confident for the future, understanding the potential pitfalls of burnout, feeling mentally well, and a partial RTW. *Distal outcomes* are outcomes not emerging during the intervention itself, including a full RTW, reduced burnout complaints, and experiencing walking in nature as an everyday resource for one’s health and wellbeing.

**Table 3:** Key outcomes triggered by the implementation of the six intervention elements and change process.

Proximate outcomes	Intermediate outcomes	Distal outcomes
Becoming a mindful owner of one’s thoughts	Regaining feeling of control over the rehabilitation process	Experiencing walking in nature as an everyday resource
Becoming physically active	Utilizing social support from employers and employees	Feeling no burnout complaints
Experiencing success	Applying new (pro-active) coping strategies	Full return to work
Feeling relaxed	Feeling meaningfulness in (working) life	
	Feeling confident for the future	
	Understanding potential pitfalls	
	Feeling mentally well	
	Partial return to work	

## Discussion

The present study aimed to develop an intervention and evaluation model of outdoor therapy to enhance our understanding of how outdoor therapies may facilitate the rehabilitation process after burnout, as well as to explore how to empirically evaluate such an outdoor intervention. We used the generic CPO model (Fridrich et al., 2015) and the generic BRM (Fridrich et al., 2015; Pijpker et al., 2021; Van Dam, 2021) as an overarching deductive frame. We then inductively identified key outdoor intervention elements and examined how the implementation of these elements may enhance GRRs in various rehabilitation processes and the resulting outcomes. We showed that outdoor therapy comprises six key outdoor intervention elements: (1) physical activity, (2) reconnecting body and mind, (3) nature metaphors, (4) creating relationships, (5) observing natural interactions, and (6) experiential learning. We also showed that the implementation of these elements strongly depends on the context of the client (e.g., rehabilitation phase), therapy (e.g., privacy issues), and therapist (e.g., number of clients per day), meaning that elements cannot be predetermined to belong to rehabilitation phases and outcomes. Finally, we identified various indicators of these contextual factors, which may facilitate the evaluation of outdoor therapy.

### Scientific Implications

To our knowledge, this is the first time an intervention and evaluation model of outdoor therapy for the rehabilitation after burnout has been empirically developed. Although previous observational and experimental studies have focused on the efficacy of various outdoor burnout therapy interventions (Annerstedt and Währborg, 2011; Sahlin et al., 2014, 2015), none of them aimed to unravel the “black box” of how such outdoor interventions may work. The present study complements the existing bank of knowledge by inductively investigating how the implementation of key outdoor intervention elements—in combination with the context of the client, therapy, and therapist—may facilitate rehabilitation after burnout.

Previous studies primarily focused on measuring burnout complaints and RTW when conducting effect evaluations (Annerstedt and Währborg, 2011; Stigsdotter et al., 2018); however, these are distal outcomes, requiring months to observe changes (Kant et al., 2004; de Vente et al., 2015; Roelen et al., 2015; Pijpker et al., 2020). We identified a range of proximal and intermediate outcomes that may emerge during and after the therapy sessions. Future studies are therefore encouraged to measure a combination of proximal (e.g., feeling mindful), intermediate (e.g., coping strategies), and distal (e.g., RTW) outcomes. Since the generic BRM is based on the salutogenic model of health (Pijpker et al., 2021), we were able to show how the key outdoor intervention elements may enhance certain GRRs in various rehabilitation phases. Our conceptual and empirical work suggests that outdoor therapy may enhance clients’ feelings of control over their rehabilitation (i.e., an overarching GRR in the BRM), which may in turn strengthen their SOC in the long term. Related to this, the social support clients experienced in our study may be another GRR enhancing SOC levels, as earlier studies have shown that SOC and social support strengthen each other, especially during negative life events (Srensen et al., 2011). Von Lindern et al. (2017) suggest nature can restore temporarily depleted physical and mental resources, potentially even temporarily depleted SOC levels (von Lindern et al., 2017); thus, it would be worthwhile to explore whether nature becomes an everyday GRR for clients after their therapy sessions have ended, as our study suggests.

Our study shows that the therapist–client relationship is an important prerequisite for the overall therapy experience. This complements other studies that concluded that relationship factors (e.g., empathy) show a consistent and moderate impact on CBT interventions (Keijsers et al., 2000). Our results suggest that outdoor therapy may facilitate more stronger relationships between clients and their therapists than indoor therapy, which can be attributed to walking side-by-side in the familiar outdoor environment and the natural flow of conversations outdoors. Earlier studies confirm our observation that outdoor therapy may indeed enhance the relationship between clients and their therapist (Cooley et al., 2020). The present study also partly aligns with the recent meta-synthesis of various outdoor therapies by Cooley et al. (2020); for example, nature metaphors and observing how the client interacts with natural elements were identified as key aspects of outdoor therapies in general in their analysis. We complement their findings by showing how the implementation of key outdoor intervention elements can be linked to the recovery process of a specific group of clients (in this case, burned-out employees).

We also showed that physical activity and experiential learning are essential elements of outdoor therapy for employee burnout, which makes sense as it is known that being physical active outdoors alleviates stress (Hartig et al., 2014) and that rehabilitation entails a process of “learning by experiencing” (Pijpker et al., 2021; Van Dam, 2021). Concerning our observation that physical activity in the outdoors enhances one’s capacity to think creatively aligns with earlier experimental studies showing that walking outdoors in nature opens up a free flow of ideas stronger than indoor walking (Oppezzo and Schwartz, 2014; Plambech and Van Den Bosch, 2015). Related to this, our study suggests that experiencing nature enables clients to get closer to their inner feelings and think more positive than being in urban or indoor environments, this has been confirmed by several experimental studies showing that spending time in nature reduces people’s focus on negative aspects of one’s self (Bratman et al., 2015, 2021). In turn, being able to get close to one’s inner feelings, think creative and positive are important processes in the rehabilitation process after burnout (Pijpker et al., 2021; Van Dam, 2021).

We identified a range of indicators related to the context of the client, therapy, and therapist, thereby enabling the further examination of how these contextual factors may influence rehabilitation after burnout. Our intervention and evaluation model of outdoor therapy can therefore be used to guide context-sensitive evaluation studies, further unraveling the “black box” of effective elements in outdoor therapy. Although our study explicitly focused on taking clients outdoors into nature as a complementary approach to conventional burnout rehabilitation therapy, the same approach may be feasible to support the recovery process for other psychological disorders like depression or anxiety.

Although our study suggests that outdoor therapy potentially can restore or enhance SOC levels, as well as strengthening work-related GRRs, such as learning and applying new coping strategies, the work environment may still be triggering the reoccurrence of burnout. For example, key work-related stressors, such as workload, work pressure, role conflict, and role ambiguity (Lee and Ashforth, 1996; Alarcon, 2011), and GRRs, such as job support and social support, are not addressed *via* outdoor therapy as these require more structural interventions in the workplace (Pijpker et al., 2020). Therefore, it is recommended to expand outdoor therapy with interventions in the workplace to reduce stressors and enhance and strengthen GRRs and SOC levels (Pijpker et al., 2018, 2021).

Finally, the present study showed how the implementation of the six intervention elements could support the rehabilitation process after burnout, depending on the role of the discrete context, such as the chosen natural environment. However, we were not able to explore the role of different types of natural environments or landscape types in relation to the implementation of intervention elements and the rehabilitation process, which offers an opportunity for future studies. For example, in their pilot study among people with stress-related exhaustion, Sonntag-Öström et al. (2011) explored the perceived restorative effects of various forests settings for recovery from stress, showing that forest settings with light were identified as most beneficial (a GRR in the salutogenic model). Besides being exposed to the natural environment, the “dosage” of time being in nature matters as well; for example, a recent study showed that 120 min per week yields the optimum effect for our general health and wellbeing (White et al., 2019). Therefore, future studies are encouraged to examine which natural environments or landscape types and duration of nature exposure are perceived as most beneficial for the rehabilitation after burnout in outdoor therapy.

### Study Limitations and Strengths

The present study has multiple limitations and strengths that should be taken into account when interpreting the results. First of all, selection bias was caused by recruiting therapists and clients who all (to a certain extent) believe in the healing effects of nature. This is, however, also a strength, as we explicitly asked what worked for them and how. Nevertheless, we were still able to identify multiple diverging experiences about the role of outdoors and nature in the therapy and rehabilitation process. A second limitation concerns the very small sample size of clients and therapists, which means that the results should be interpreted with caution regarding their generalizability. However, since we aimed to exploratively and inductively develop an intervention and evaluation model based on a deductively derived generic frame, the triangulation of primary and secondary data sources were deemed appropriate and generally known as valid and reliable methods when using case study designs (Yin, 2013). The major strength and aim of this study are that our model can guide future context-sensitive evaluation studies to test our model among a larger group of clients. A third limitation is that all primary data collection was held online due to the COVID-19 measures, which made it impossible to conduct face-to-face interviews; however, online interviews are not intrinsically less valid or reliable than real-life interviews (Pocock et al., 2021). A fourth limitation is that we did not explicitly compare indoor therapy versus outdoor therapy, making it hard to claim that the intervention elements identified in our study are fully mutually exclusive of those for indoor therapy. Future studies could compare the efficacy of indoor versus outdoor therapies to understand whether and why outdoor therapy is more effective than indoors (or not). Finally, although the combination of our deductive and inductive approaches and the variety of data sources has yielded a rich understanding of our exemplary outdoor therapy for employee burnout, the model also offers a research opportunity to study whether and how the intervention elements may work for other psychological disorders.

## Conclusion

The present study aimed to develop an intervention and evaluation model of outdoor therapy for employee burnout. We identified the key outdoor intervention elements (i.e., physical activity; reconnecting body and mind; nature metaphors; creating relationships; observing natural interaction; and experiential learning) and suggest that their successful implementation strongly depends on the discrete context of the client, therapy, and therapist. We identified which GRRs may be strengthened by the implementation of the outdoor intervention, thereby facilitating the rehabilitation process after burnout. Finally, we identified a range of indicators for the process and outcome evaluation of outdoor therapy for employee burnout. Future studies are encouraged to use our intervention and evaluation model as a guide for a context-sensitive evaluation of outdoor therapy for employee burnout and other client and patient groups.

## Data Availability Statement

The data generated and analyzed during the current study are not publicly available in accordance with protection of confidentiality and privacy, but are available from the corresponding author on reasonable request. Requests to access the datasets should be directed to roald.pijpker@wur.nl.

## Ethics Statement

The methodology for this study was approved by the Social Sciences Ethics Committee (Wageningen University and Research) on June 13, 2019. Prior to the interviews, the first author emailed the participants to inform them about the aims of the study. The researchers were aware that interviews with former clients involved a potential risk of traumatization by asking people to revisit what they had experienced before. The participants were informed about this possibility and were told that they could contact the interviewer and occupational doctor at any time after the interview. All participants provided signed informed consent before the interviews took place. Since all interviews took place online due to the COVID-19 restrictions, they again provided consent before starting the interview.

## Author Contributions

RP, EV, LV, MK, and GB: conceptualization, methodology, validation, and writing—review and editing. RP: formal analysis, writing—original draft preparation, visualization, and project administration. EV, LV, MK, and GB: supervision. All authors contributed to the article and approved the submitted version.

## Funding

This study was funded by the internal funds of two chair groups—Health and Society and Rural Sociology (Center for Space, Place and Society)—at Wageningen University & Research in the Netherlands.

## Conflict of Interest

The authors declare that the research was conducted in the absence of any commercial or financial relationships that could be construed as a potential conflict of interest.

## Publisher’s Note

All claims expressed in this article are solely those of the authors and do not necessarily represent those of their affiliated organizations, or those of the publisher, the editors and the reviewers. Any product that may be evaluated in this article, or claim that may be made by its manufacturer, is not guaranteed or endorsed by the publisher.

## Footnotes

^1^ https://www.debuitenpsychologen.nl/

^2^IP, intervention protocol; CL, client; OT, outdoor therapist; ID, intervention developers. The numbers refer to the document and quotation number.
